# Multiply clustered gold-based nanoparticles complexed with exogenous pDNA achieve prolonged gene expression in stem cells

**DOI:** 10.7150/thno.34487

**Published:** 2019-07-09

**Authors:** Se Won Yi, Ji Sun Park, Hye Jin Kim, Jung Sun Lee, Dae Gyun Woo, Keun-Hong Park

**Affiliations:** 1 Department of Biomedical Science, College of Life Science, CHA University, 6F, CHA Biocomplex, Sampyeong-Dong, Bundang-gu, Seongnam-si, 13488, Republic of Korea.; 2 CHA Advanced Research Institute, 7F, CHA Biocomplex, Sampyeong-Dong, Bundang-gu, Seongnam-si, 13488, Republic of Korea.

**Keywords:** multiple cluster, prolonged gene delivery, gold nanoparticle, heparin, electrostatic interaction, hMSCs, chondrogenic differentiation

## Abstract

Development of a stable and prolonged gene delivery system is a key goal in the gene therapy field. To this end, we designed and fabricated a gene delivery system based on multiply-clustered gold particles that could achieve prolonged gene delivery in stem cells, leading to improved induction of differentiation.

**Methods**: Inorganic gold nanoparticles (AuNPs) underwent three rounds of complexation with catechol-functionalized polyethyleneimine (CPEI) and plasmid DNAs (pDNAs), in that order, with addition of heparin (HP) between rounds, yielding multiply-clustered gold-based nanoparticles (mCGNPs). Via metal-catechol group interactions, the AuNP surface was easily coordinated with positively charged CPEIs, which in turn allowed binding of pDNAs.

**Results**: Negatively charged HP was encapsulated with the positive charge of CPEIs via electrostatic interactions, making the NPs more compact. Repeating the complexation process yielded mCGNPs with improved transfection efficiency in human mesenchymal stem cells (hMSCs); moreover, these particles exhibited lower cytotoxicity and longer expression of pDNAs than conventional NPs. This design was applied to induction of chondrogenesis in hMSCs using pDNA harboring SOX9, an important chondrogenic transcription factor. Prolonged expression of SOX9 induced by mCGNPs triggered expression of chondrocyte extracellular matrix (ECM) protein after 14 days, leading to more efficient chondrogenic differentiation *in vitro* and *in vivo*.

## Introduction

Gene delivery by non-viral vectors is a promising biomedical strategy that provides a safe and effective approach for treating various diseases by intentionally altering gene expression levels 1, 2. From this standpoint, successful clinical application of gene-based therapy relies on efficient gene delivery vehicles 3, 4. Multiple trials have demonstrated that the efficiency of gene transfer into cells can be improved by modifying conventional non-viral vectors 5, 6. One important aspect of gene delivery is the duration of expression of the delivered genes 7-9. Permanent or prolonged gene expression could obviate the need for additional therapy, or at least decrease the frequency of return to the clinic, emphasizing the importance of treatments that can achieve long-term gene expression 10. To address these needs, we have designed novel gene delivery vehicles and applied them to stem cells, resulting in better control of differentiation into specific cell types.

In this study, we explored the use of gold-based nanoparticles (AuNPs) in such systems. AuNPs are important tools in nanotechnology due to their ease of surface functionalization 11, biocompatibility, chemical stability, and detectability by surface plasmon resonance (SPR) 12; consequently, they are promising materials for biomedical research 13-15. Accordingly, AuNPs have been hybridized with other biomaterials for use in novel applications, including gene therapy 16-18. In this context, the combination of AuNPs with polymer or hydrogels (hyaluronic acid 19, 20, heparin 21, 22, chitosan 23, etc), followed by cohesion, has attracted a great deal of attention.

In previous studies, gold nanoparticles of 80 ~ 150nm size, which is easy to transfer into cells, were coated with DOPAbPEI to transfer the gene, but there was a limitation in expressing the gene for a long time. Therefore, we have tried to produce transporter capable of gene expression for a long time.

We found that in contrast to previously designed AuNPs, small AuNPs (5-20nm) could be assembled with polymers and gels to form core-nucleated gold nanoclusters.

Once too small a gold nanoparticle (> 20 nm) was not suitable as an intracellular delivery carrier, heparin was used as a polymer for coagulation formation. In addition, to make cation carriers, gold nanoparticles were adsorbed on the surface of gold nanoparticles, and dopa-bPEI, which has many cations, was bonded. The 3,4-dihydroxyphenylalanine of DOPA-conjugated PEI was easily adsorbed on gold nanoparticles (~ 5 nm), and the cationic polymer, bPEI, was well linked to the gene. The resultant structures were not only the ideal size for gene delivery (80-250 nm), but also provided additional functions that promote prolonged expression of the delivered genes, thereby improving the overall efficacy of gene therapy. Small gold nanoparticles (~ 5 nm) will aggregate with heparin and have a size (> 150 nm) suitable for entry into the cell. In addition, clusters are degraded in cells, and gold nanoparticles (~ 5 nm) are loosened one by one, and they function as gene carriers. In other words, mCGNPs contain large amounts of genes in the core, and they have an appropriate size and shape that is easy to enter into cells. Subsequently, each transporter is slowly released in the cell to efficiently deliver the gene.

**Scheme 1:**
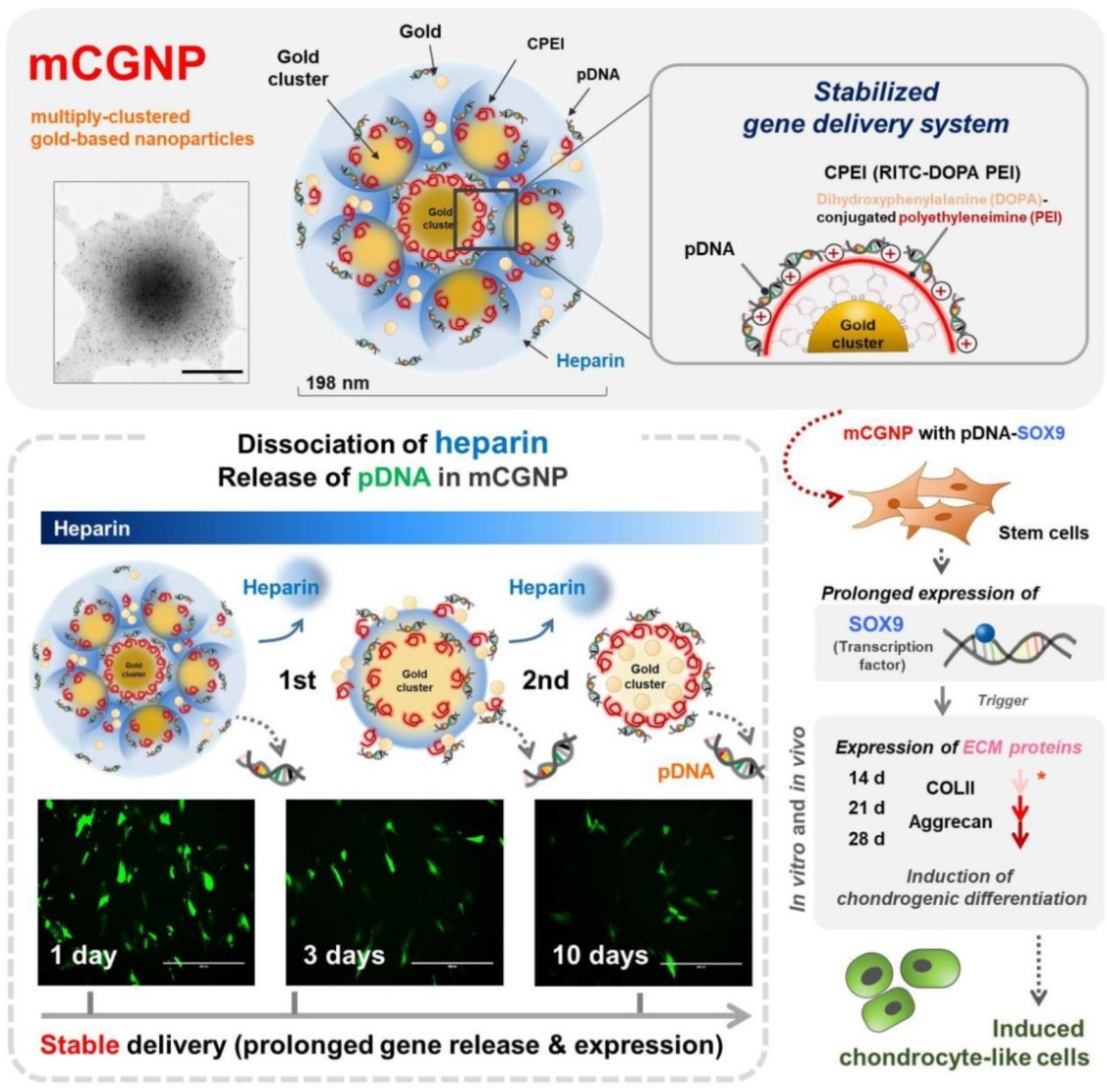
** Illustration of multiply-clustered gold-based nanoparticles (mCGNPs) fabricated by repeated complexation of gold nanoparticles (AuNPs), catechol-functionalized polyethyleneimine (CPEI), and pDNA with heparin (HP), resulting in prolonged gene delivery and expression *in vitro* and *in vivo*.** In this study, this system was used to induce stem cells to undergo chondrogenic differentiation by introducing pDNA-SOX9. Prolonged expression of SOX9 triggered expression of chondrogenic ECM proteins, including collagen type II (COLII) and aggrecan (AGG), from the early stage, thereby promoting and accelerating chondrogenic differentiation.

Polyethyleneimine (PEI), a vital factor in gene delivery 24-26, can be modified with 3,4-dihydroxyphenylalanine (DOPA), which contains catechol groups, yielding catechol-functionalized PEI (CPEI). Catechol groups interact with metallic materials, including iron oxide, gold, and silver 27, 28. Thus, CPEI can coordinate onto the surface of AuNPs, promoting complexation with plasmid DNA (pDNA). As illustrated in Scheme 1, AuNPs, CPEI, and pDNAs undergo three rounds of complexation. Between the rounds, heparin (HP), a negatively charged polysaccharide 21, 29, is added and encapsulated in the complexes, providing a more compact structure. Polysaccharides are natural molecules with advantages for theranostics, including diversity of size and charge, abundance, biodegradebility, and low toxicity *in vivo* 30, 31. In particular, as materials for NPs, they are increasingly being used to control the release of molecular payloads 22, 32, 33.

By repeating the complexation process, multiply-clustered gold-based nanoparticles (mCGNPs) can be fabricated to serve as nanoscale gene delivery vehicles. For a comparative evaluation of the mCGNPs, we also fabricated singly-clustered gold- based nanoparticles (sCGNPs), which contain all of the aforementioned components except for HP and are formed by a different complexation method. Comparison of uptake efficiency, gene expression efficiency, and expression patterns of the delivered genes revealed that mCGNPs were more effective than sCGNPs for stable and long-term gene delivery.

Finally, pDNA harboring SOX9 (SRY-Box9) was incorporated into mCGNPs, and the resultant particles were used to induce human mesenchymal stem cells (hMSCs) to undergo chondrogenic differentiation *in vitro* and *in vivo*. SOX9 acts as a master regulator of chondrogenesis by promoting expression of extracellular matrix (ECM) proteins, including *Col2a1* and aggrecan (AGG) 34-36. Because SOX9 is a key chondrogenic transcription factor, it must be expressed in most periods of chondrogenesis 37-39. Therefore, to promote chondrogenic differentiation of cells, pDNA-SOX9 complexed to mCGNPs must be released efficiently and expressed as long as possible. We achieved longer-term expression of SOX9 with mCGNPs than with sCGNPs. Longer-term expression of this transcription factor improved and accelerated induction of chondrogenic differentiation.

## Methods

### Materials

AuNPs (5 nm in water, EM.GC5) were purchased from BBI Solutions (Newport, Wales, UK). 3-(3,4- Dihydroxyphenyl) propionic acid (DPA), bPEI (25 kDa), and RITC were purchased from Sigma-Aldrich (St. Louis, MO, USA). Fluorescein-conjugated HP (H7482) was purchased from Invitrogen (Carlsbad, CA, USA).

### Synthesis of CPEI

DOPA-conjugated PEI was synthesized by the conventional carbodiimide reaction as previously described 17. The fluorescent dye RITC (λ_ex_: 562 nm; λe_m_: 583 nm) was chemically conjugated to the amine groups of PEI via the isocyanate-amine reaction, yielding CPEI.

### Fabrication of sCGNPs and mCGNPs

AuNPs ~5 nm in diameter were coated with CPEI via the catechol-mediated interaction between the AuNP surface and the catechol groups of DOPA. These cationic NPs were complexed with anionic pDNA. Total amount of AuNP, CPEI (5µg~ 15µg), and pDNA (3µg) were mixed at once, yielding sCGNPs. On the other hands, the sequential assembly process of complexation of 1/3 of total amount, AuNP, CPEI (1.67µg ~ 5µg), and pDNA (1µg), was repeated three times, and HP (5µg ~ 10µg) was encapsulated between each round, yielding mCGNPs.

### Characterization of sCGNPs and mCGNPs

The hydrodynamic diameter and surface charge of NPs were measured with a Zetasizer Nano ZS (Malvern Incorporated, Malvern, UK). NPs were dispersed in ultra-pure water, and 20 measurements were conducted per sample. The morphology and distribution of NPs was visualized by TEM (H-7600, Hitachi, Tokyo, Japan) and AFM (NanoWizard II, JPK Instruments, Berlin, Germany). Samples were coated onto carbon film on 200 mesh copper grids (Electron Microscopy Science, Hatfield, PA, USA), dried overnight at room temperature, and visualized by TEM (ARM200F, JEOL, Tokyo, Japan). The levels of AuNP, CPEI, and HP in sCGNPs and mCGNPs were determined by various tools based on the physical and chemical properties of each component. AuNPs were characterized using a UV-vis spectrometer (TECAN PRO 200, Männedorf, Switzerland) in the wavelength range from 450 to 700 nm. CPEI and HP were detected on a fluorescence plate reader (TECAN PRO 200) using a ChemiDoc imaging system (BR170-8265, Bio-Rad Laboratories, Hercules, CA, USA), after which the fluorescence intensity was calculated.

### Dissociation of HP from mCGNPs

FL-HP (H7482, Invitrogen) was used to detect HP associated with mCGNPs. mCGNPs complexed with FL-HP were suspended in phosphate-buffered saline (PBS, PH 7.4) and incubated in 5 % CO_2_ at 37 °C. Samples were taken at pre-determined time intervals (1, 2, 3, 7, and 10 days) and centrifuged for 30 min. The supernatant was collected and subcutaneously injected into BALB/c nude mice, and fluorescence was detected (λ_ex_: ~480 nm; λ_em_: ~520 nm) on a Xenogen instrument (IVIS imaging system 200, PerkinElmer, Waltham, MA, USA). The animal study was approved by the Institutional Animal Care and Use Committee (IACUC) of CHA. Fluorescence intensity of the collected supernatant was analyzed on a microplate reader (TECAN PRO 200).

### Release of loaded DNA from sCGNPs and mCGNPs

sCGNP and mCGNP complexes were suspended in PBS (PH 7.4) and incubated in 5 % CO_2_ at 37 °C. Samples were taken at various time intervals (1, 2, 3, 7, and 10 days) and centrifuged at 13,000 rpm for 30 min. Supernatants were analyzed to determine the concentration of released DNA. The concentration of released pDNA was quantified by the PicoGreen assay (P11496, Invitrogen). pDNA solution from CGNPs (sCGNPs and mCGNPs) was diluted in TE buffer (10 mM Tris-HCl, 1 mM EDTA, PH 7.5). An equal volume of Quant-iT™ PicoGreen reagent was added to the samples and incubated for 2 to 5 min at room temperature in the dark. Fluorescence was measured on a microplate reader (TECAN PRO 200) at an excitation wavelength of 480 nm and an emission wavelength of 520 nm and detected using a ChemiDoc XRS system (BR170-8265, Bio-Rad Laboratories).

### Gel retardation assay

To confirm that the NPs were capable of binding cargo genes, gel retardation assay was performed and visualized on a ChemiDoc imaging system (BR170-8265, Bio-Rad Laboratories, Hercules, CA, USA).

### Cell culture

hMSCs isolated from the bone marrow of a 20-year-old man were purchased from Lonza (PT-2501, Lonza, Walkersville, MD, USA). hMSCs were cultivated in DMEM high glucose medium (SH30022) supplemented with 10 % fetal bovine serum and 1% antibiotic-antimycotic solution in a humidified 5 % CO_2_ incubator at 37 °C. The culture medium was replaced every 2-3 days. All experiments were performed with cells at passages 5-7.

### Gene transfection of hMSCs using sCGNPs and mCGNPs

hMSCs were transfected with pDNAs, using the optimal composition and concentration of sCGNPs and mCGNPs, in serum-free medium for 20 min, followed by incubation for 4 hours.

### Cytotoxicity studies of sCGNPs and mCGNPs in hMSC

Cytotoxicity was evaluated using the Live/Dead assay (Invitrogen). hMSCs treated with sCGNPs or mCGNPs were incubated in a solution containing 2 µM calcein AM (to stain live cells) and 4 µM EthD-1 (to stain dead cells) and visualized by fluorescence microscopy (EVOS FL, Thermo Fisher, Waltham, MA, USA). The state of cells treated with NPs was confirmed by FACS (Guava Technologies, Hayward, CA, USA).

### Creation of articular cartilage defected rat models and mCGNP application

Eight-week-old Sprague Dawley (SD) rats were used in this study. After para-patellar arthrotomy, the patellar groove was exposed by laterally dislocating patella and partial cartilage defect along the groove by the biopsy punch (1.0mm diameter, BP-10F, Kai Corporation, Tokyo, Japan). To establish the mode, a defect (200 µm × 9 mm^2^; depth × area) was introduced into the knee (i.e., the site of patellar groove). Subsequently, prepared pellets of hMSCs (non-treated, sCGNP-treated, or mCGNP-treated) were transplanted at the defect sites in fibrin gel (Beriplast-P Combi-set, CSL Behring, Marburg, Germany). After the patella was relocated, the muscle and skin were sutured with a black silk 5-0 (SK526, Ailee Company Limited, Busan, Korea). At 1, 3, and 6 weeks after transplantation, three rats (two legs, n=6) from each group were sacrificed, the transplanted pellets were collected and the extent of repair was evaluated.

### Reverse transcription-polymerase chain reaction (RT-PCR)

For analysis of mRNA, total RNA was extracted using TRIzol (Thermo Fisher Scientific), and quality and concentration were determined using a spectrophotometer (NanoDrop 2000, Thermo Fisher Scientific). Total RNA (500 ng) was reverse-transcribed according to the manufacturer's protocol (M-MLV Reverse Transcriptase, Invitrogen). PCR reactions were performed using G-Taq PCR Mastermix (CMT7001, Cosmogenetech, Seoul, Korea) under conditions appropriate for each primer. PCR products were stained with ethidium bromide (EtBr) and confirmed on agarose gels visualized under UV light.

### Western blotting analysis

For analysis of protein, total protein was extracted using radioimmunoprecipitation (RIPA) buffer. Cell lysates (40 µg) were analyzed on 10% SDS-PAGE gels. Blots were incubated with primary antibodies and HRP-conjugated secondary antibodies (Bio-Rad); buffers contained 2.5% skim milk. Signals were detected on films using enhanced chemiluminescence (Amersham Pharmacia; GE Healthcare, Pittsburgh, PA, USA).

### Immunofluorescence analysis

The pellets of hMSCs cultured in 3D systems and collected from defected cartilage of rats were fixed with 4% paraformaldehyde, embedded in optimal cutting temperature compound (TISSUE-TEK 4583; Sakura Finetek Inc., Torrance, CA, USA), and sliced into sections (10 µm thick) using a cryotome (HM 525; Thermo Fisher Scientific). Immunofluorescence was performed under humidified conditions using primary antibodies against SOX9 (AB5335, Rb, Abcam) and COLII (MAB 1330, Ms, Millipore). Thereafter, samples were stained with fluorescently labeled secondary antibodies (1:500; Thermo Scientific), incubated with DAPI for 10 min to stain the nucleus, and visualized by fluorescence confocal microscopy (LSM 880 META, Zeiss).

## Results and Discussion

### Characterization of sCGNPs and mCGNPs

**Figure 1:**
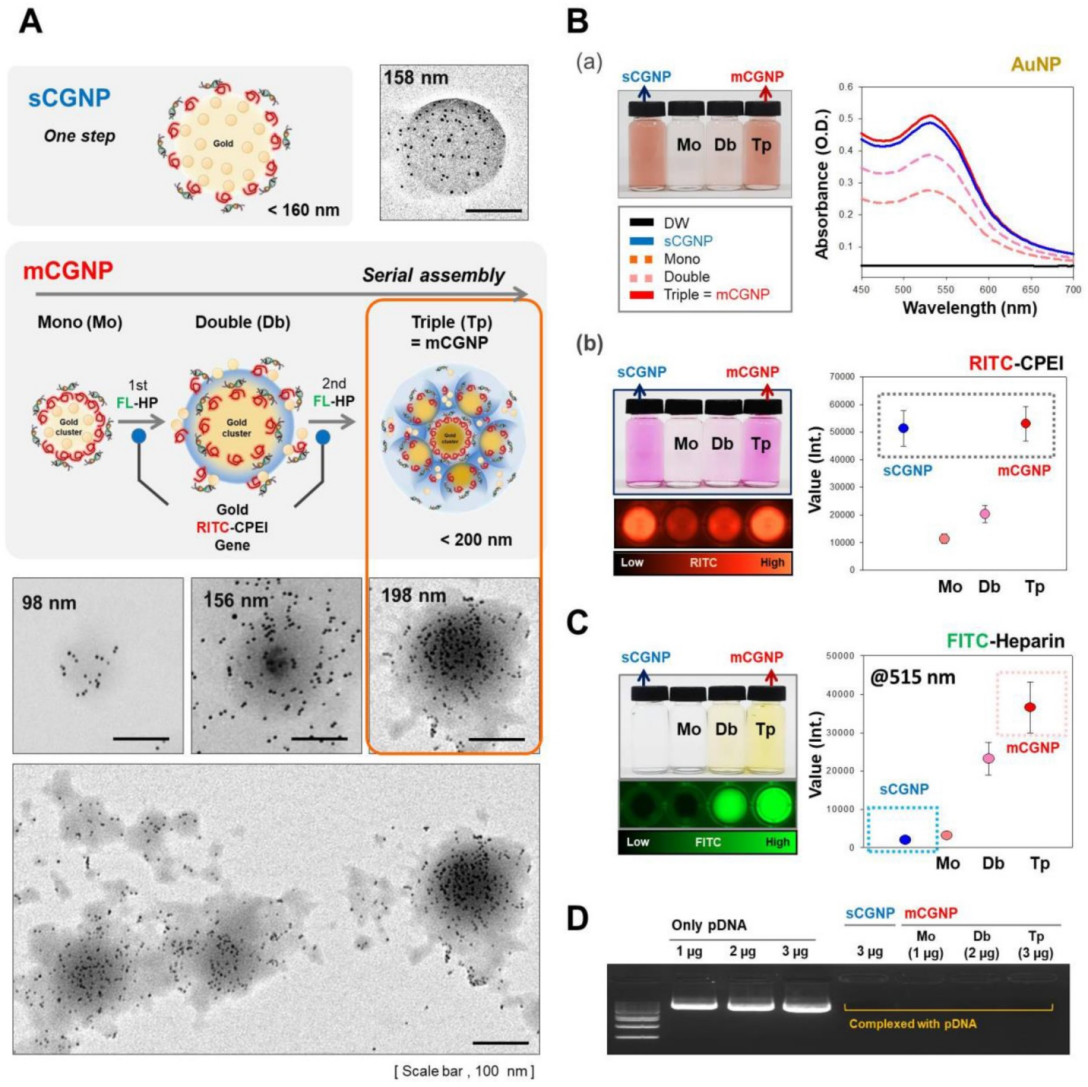
** Characterization of sCGNPs and mCGNPs.** Morphologies and sizes of sCGNPs and mCGNPs, determined by transmission electron microscopy, with brief schematic features of the fabrication process (A). Composition and amount of AuNPs and RITC-CPEI was determined by measuring absorbance on a plate reader (B). Composition and amount of heparin (HP) conjugated to FITC (FL-HP) was determined by measuring fluorescence (C). Finally, complexation of pDNA was confirmed by gel retardation assay (D). Scale bar, 100 nm.

We sought to fabricate mCGNPs by multiple rounds of complexation with HP, with the hope of forming more compact and layered structures that could achieve prolonged delivery of exogenous pDNA in transfected cells. In parallel, we synthesized gold clustered NPs with a single round of complexation, without HP (sCGNPs), using the same amount of AuNPs, CPEI, and pDNA as in the mCGNP system. As shown in fig. 1A, mCGNPs were fabricated in three steps, consisting of repeated rounds of complexation of AuNPs, CPEI, and pDNA in that order. Between the steps, HP was added by ionic complexation with CPEI. By contrast, sCGNPs were synthesized in one step. Intermediate products, including mono-clusters (Mo) and double clusters (Db), were produced at the first and second step of complexation; finally, after the third step, the triple clusters called mCGNPs were obtained. The sizes of Mo, Db, and Tp (mCGNPs) were confirmed by dynamic light scattering (DLS) to be 98, 156, and 198 nm, with positive charge (Figure S1) indicating that they were of the proper size to serve as nano-carriers. As shown in the transmission electron microscopy (TEM) image, the NP structures in each of the three steps were getting tightened, and ultimately condensed mCGNPs with nucleated AuNPs were obtained (Figure 1A). DLS analysis and atomic force microscopy (AFM) revealed that the size distribution of mCGNPs was monodisperse (Figure S2A, B). The stability of the prepared mCGNP and sCGNP was confirmed at 1, 3, 7, 10, and 14 days in the aqueous solution, and gradually precipitated from day 7 (Figure S3).

Except for HP, sCGNPs and mCGNPs have the same components (Au (gold) of AuNP and N (nitrogen) of CPEI), as confirmed by elemental mapping by energy-dispersive X-ray spectroscopy (EDS) (Figure S4). First, we confirmed the relative amounts of components, including AuNPs, CPEI, and pDNA. sCGNPs and mCGNPs contained the same amount of AuNPs (Figure 1B-a), and both types of particles had absorbance peaks at a wavelength of about 540 nm. As the complexation steps of mCGNPs progressed, the absorbance peak increased in intensity and shifted slightly to the right, indicating that the particles got bigger as the AuNP content increased. Also, sCGNPs and mCGNPs contained the same amount of CPEI (Figure 1B-b). CPEI was detected with a fluorescence detector (ChemiDoc) via coupled rhodamine-B-isothiocyanate (RITC), which yielded similar values for sCGNPs and mCGNPs. As expected, HP was detected in mCGNPs, but not in sCGNPs. HP was detected on a fluorescence detector using FITC-conjugated HP (FL-HP) (Figure 1C). The FITC level in mCGNPs increased with the number of complexation steps. A gel retardation assay revealed that all components were tightly associated by mutual electrostatic interactions, i.e., the pDNA was efficiently complexed within the mCGNPs (Figure 1D).

### In heparin and gene release from sCGNPs and mCGNPs

**Figure 2:**
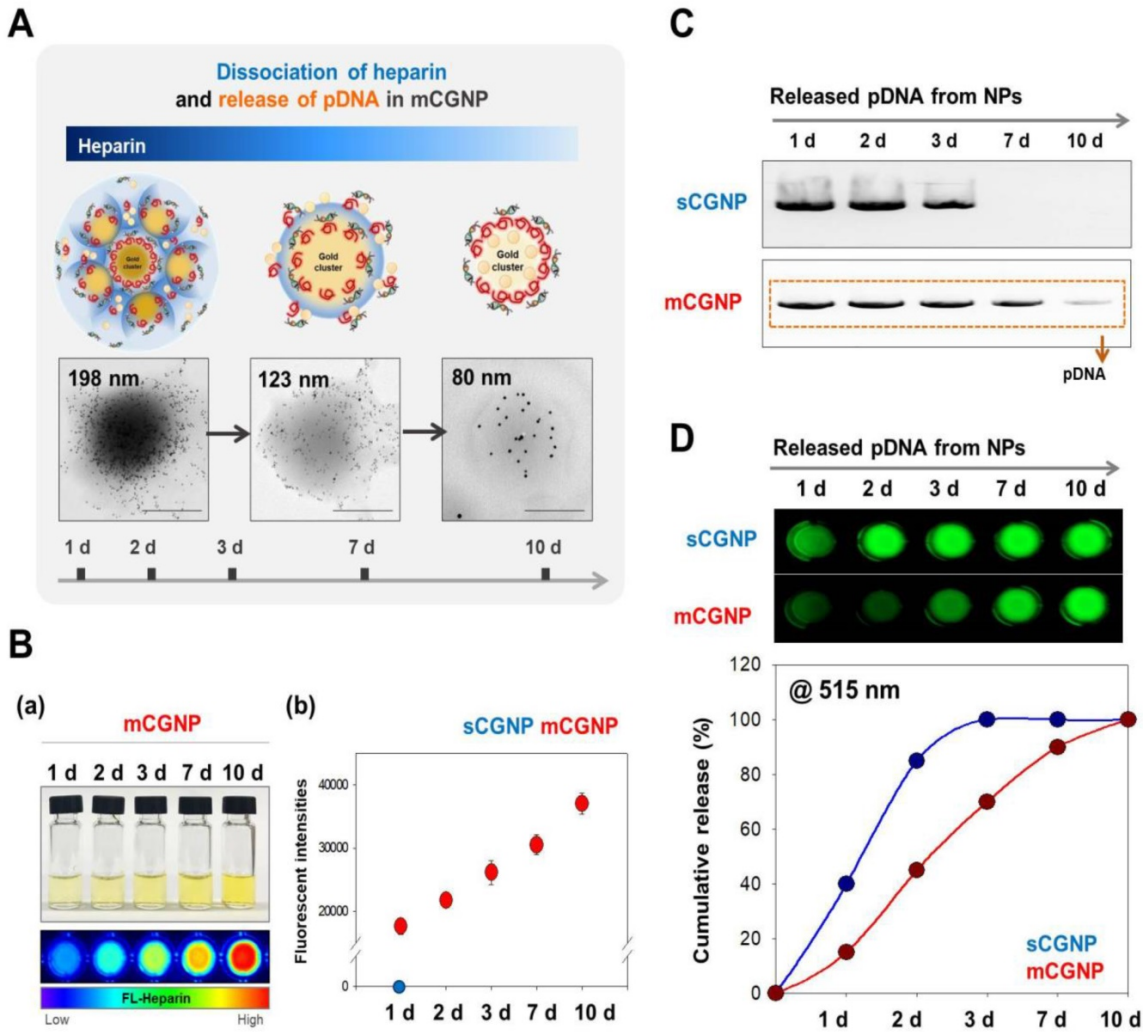
** Evaluating dissociation of heparin and release of loaded pDNA from sCGNPs and mCGNPs over 10 days.** Brief illustration and TEM image showing dissociation of heparin with release of pDNA from mCGNPs for 10 days after fabrication (A). FL-HP collected from mCGNPs on days 1, 2, 3, 7, and 10 was subcutaneously injected into the mice and detected using a Xenogen instrument (B-a). The graph (B-b) shows the detected intensity value in the mice. pDNAs released from sCGNPs and mCGNPs were collected on days 1, 2, 3, 7, and 10. Qualitative and quantitative analysis of the pattern of pDNA release from sCGNPs and mCGNPs was performed by gel retardation assay (C) with the PicoGreen assay (D). Scale bar, 100 nm.

The compact mCGNPs released pDNA due to dissociation of HP complexed with CPEI over time. Dissociation of HP and release of pDNA from mCGNPs were examined over the course of 10 days. TEM imaging confirmed that mCGNPs gradually loosened due to dissociation of HP, leading to the release of pDNA (Figure 2A). The use of FL-HP made it possible to trace or measure the relative amount of HP by confocal laser microscopy or on a microplate reader. During 10 days, released FL-HP from mCGNP was collected and detected by microplate fluorescence detector (Figure 2B-a). From days 1 to 10, FL-HP was detectable with the samples from mCGNPs, but not sCGNPs (Figure 2B-b). pDNA released from sCGNPs and mCGNPs was collected at 1, 2, 3, 7, and 10 days after complexation and subjected to gel retardation assay. Released pDNA was detectable for 10 days in samples containing mCGNPs, but only 3 days in those containing sCGNPs (Figure 2C). The same result was obtained in the PicoGreen assay (Figure 2D).

### Cytotoxicity and dissociation from sCGNPs and mCGNPs

**Figure 3:**
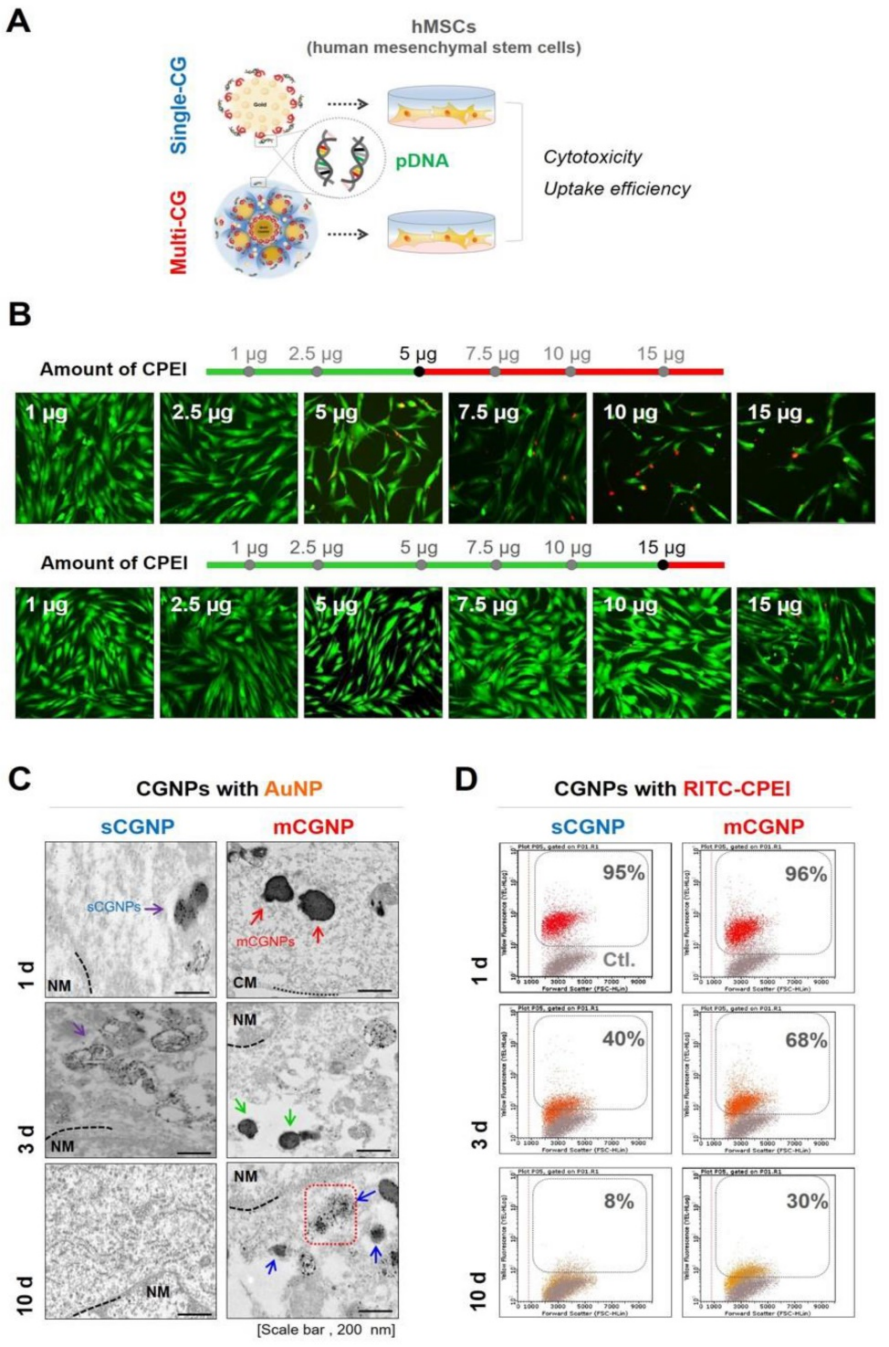
** Cytotoxicity and dissociation of mCGNPs and sCGNPs in cells.** A brief scheme shows that human mesenchymal stem cells (hMSCs), which were transfected with CGNPs (sCGNPs and mCGNPs) complexed with pDNA, were observed for cytotoxicity and dissociation of sCGNPs or mCGNPs for 10 days after transfection (A). Optimal concentration range of CPEI in sCGNPs and mCGNPs was determined by live/dead assay (B). Images of cell TEM analysis (C) and the results of FACS analysis (D) show the gradual dissociation of AuNP from sCGNPs and mCGNPs at 1, 3, and 10 days after internalization. Scale bar, 200 nm.

To assess gene delivery activities, hMSCs were treated with sCGNPs and mCGNPs with pDNA and cultured until 10 d after transfection (Figure 3A). First, we performed live/dead assay to identify the proper concentration of CPEI in CGNP system (Figure 3B). The optimal quantity of CPEI was less than 5 µg in sCGNP system, while less than 15 µg in mCGNP system. With 7.5 µg of CPEI, the mCGNP-treated sample contained almost no dead cells (red color), whereas the sCGNP-treated sample had many more dead cells. Taken together, mCGNPs had lower cytotoxicity than sCGNPs. The cellular uptake study revealed that almost all hMSCs were taken up mCGNPs and sCGNPs as determined by FACS analysis (Figure S5A), confocal laser microscopy (Figure S5B), and TEM (Figure S5C). After internalization into cells, almost all NPs were concentrated near the nucleus, implying that both sCGNPs and mCGNPs entered cells and escaped from endosomes, ultimately drawing near the nucleus to deliver genes. Due to complexation with HP, the mCGNPs were more compact structures, resulting in sustained release of complexed pDNA and leading in turn to prolonged gene expression. After cellular uptake of mCGNP, AuNP and CPEI gradually dissociated from mCGNPs via degradation of HP, resulting in the gradual release of pDNA. TEM analysis revealed gradual dissociation of AuNPs near the nucleus until day 10 after transfection in the mCGNP-system, while until day 3 in the sCGNP-system (Figure 3C). In addition, RITC- CPEI could be traced by FACS analysis. RITC-CPEI in mCGNPs was detected until day 10 (30 % of cells) after transfection, and until day 3 in sCGNP-treated cells (Figure 3D).

To investigate the duration of gene expression in stem cells, human mesenchymal stem cells (hMSCs) were exposed to mCGNPs and sCGNPs harboring pDNA-EGFP, and then harvested 1, 3, or 10 days after transfection. On day 1, mCGNPs had higher gene expression efficiency than bPEI or sCGNP, and the expression was maintained for 10 days after transfection, but not by bPEI and sCGNP in RNA and protein level as determined by RT-PCR and western blotting (Figure S6A, B). When hMSCs were treated with mCGNPs harboring pDNA-EGFP, GFP expression was maintained for 10 days after transfection (Figure S6). Although GFP RNA, GFP protein persisted for 10 days using the mCGNP system, it was not detectable in the sCGNP system at this time point (Figure S10A, C). Protein levels, as determined by FACS, western blotting, and confocal laser microscopy, reflected the RNA levels (Figure S10B, C, D).Thus, mCGNPs achieved much more prolonged gene expression than sCGNPs, likely due to their structural features. In addition to GFP, another reporter gene, pDNA-Luciferase, was conjugated to various carriers and the efficiency of each carrier gene was confirmed on days 1, 3, 7, and 10. Like GFP, pLuc was expressed up to 10 days when combined with mCGNP (Figure S7).

### Chondrogenic differentiation using sCGNPs or mCGNPs in a 3D environment

**Figure 4:**
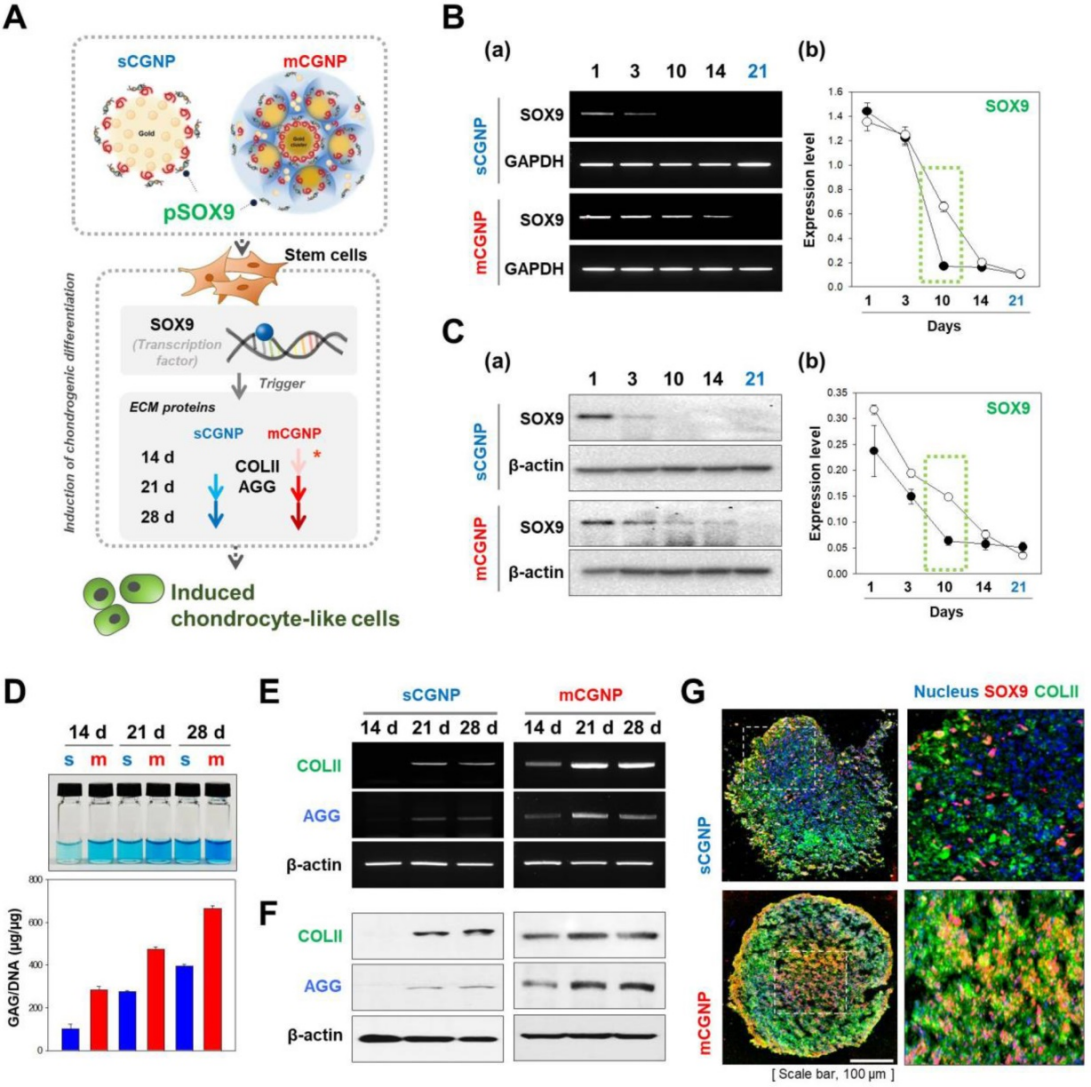
**Evaluation of chondrogenic differentiation in hMSCs treated with pDNA-SOX9 on sCGNPs or mCGNPs in a 3D environment**. Illustration showing how pDNA-SOX9 delivered by mCGNPs efficiently triggered induction of chondrogenic differentiation in hMSCs by promoting expression of extracellular matrix (ECM) proteins, including COLII and aggrecan (AGG), earlier and more effectively than the same construct delivered by sCGNPs (A). Expression of pDNA-SOX9 was evaluated for 21 days after transfection. RNA and protein levels of pDNA-SOX9 expression following delivery by sCGNPs or mCGNPs were determined by RT-PCR (B) and western blotting analysis (C), respectively. At 14, 21, and 28 days after transfection, GAG assays were performed to evaluate ECM proteins from hMSCs (D). RNA and protein levels of COLII and AGG were determined by RT-PCR (E) and western blot analysis (F), respectively. Pellets of hMSCs cultured for 21 days were sectioned, immunostained for SOX9 and COLII, and visualized by confocal laser microscopy (G). Scale bar, 100 µm.

We tested whether the mCGNP system could efficiently induce chondrogenic differentiation in hMSCs in a 3D culture system. To this end, we delivered SOX9, a key chondrogenic transcription factor, into hMSCs. mCGNPs containing pDNA-SOX9 triggered faster and more prolonged expression of ECM proteins involved in chondrogenesis, including collagen type II (COLII) and AGG, than sCGNPs (Figure 4A). Moreover, pDNA-SOX9 was expressed for longer (until day 14) when delivered on mCGNPs than on sCGNPs (Figure 4B, C). In a glycosaminoglycan (GAG) assay, ECM proteins exhibited much more prolonged expression (14 days) in the mCGNP system (Figure 4D). ECM-related genes were expressed earlier, at both the RNA and protein levels, when delivered on mCGNPs (day 14) than on sCGNPs (day 21), resulting in faster induction of chondrogenic differentiation (Figure 4E, F). Finally, we performed confocal laser microscopy to visualize the chondrocyte-related proteins SOX9 (red fluorescence) and COLII (green fluorescence) in pellets treated with sCGNPs or mCGNPs (Figure 4G). mCGNP-treated hMSC pellets contained lacunae in their centers, and expressed COLII and SOX9 evenly throughout. In addition, mCGNP-treated hMSCs pellets stained brightly with Alcian blue and Safranin O, which label proteoglycans (Figure S11).

### Effect of sCGNPs or mCGNPs in the rat model of articular cartilage defect

**Figure 5:**
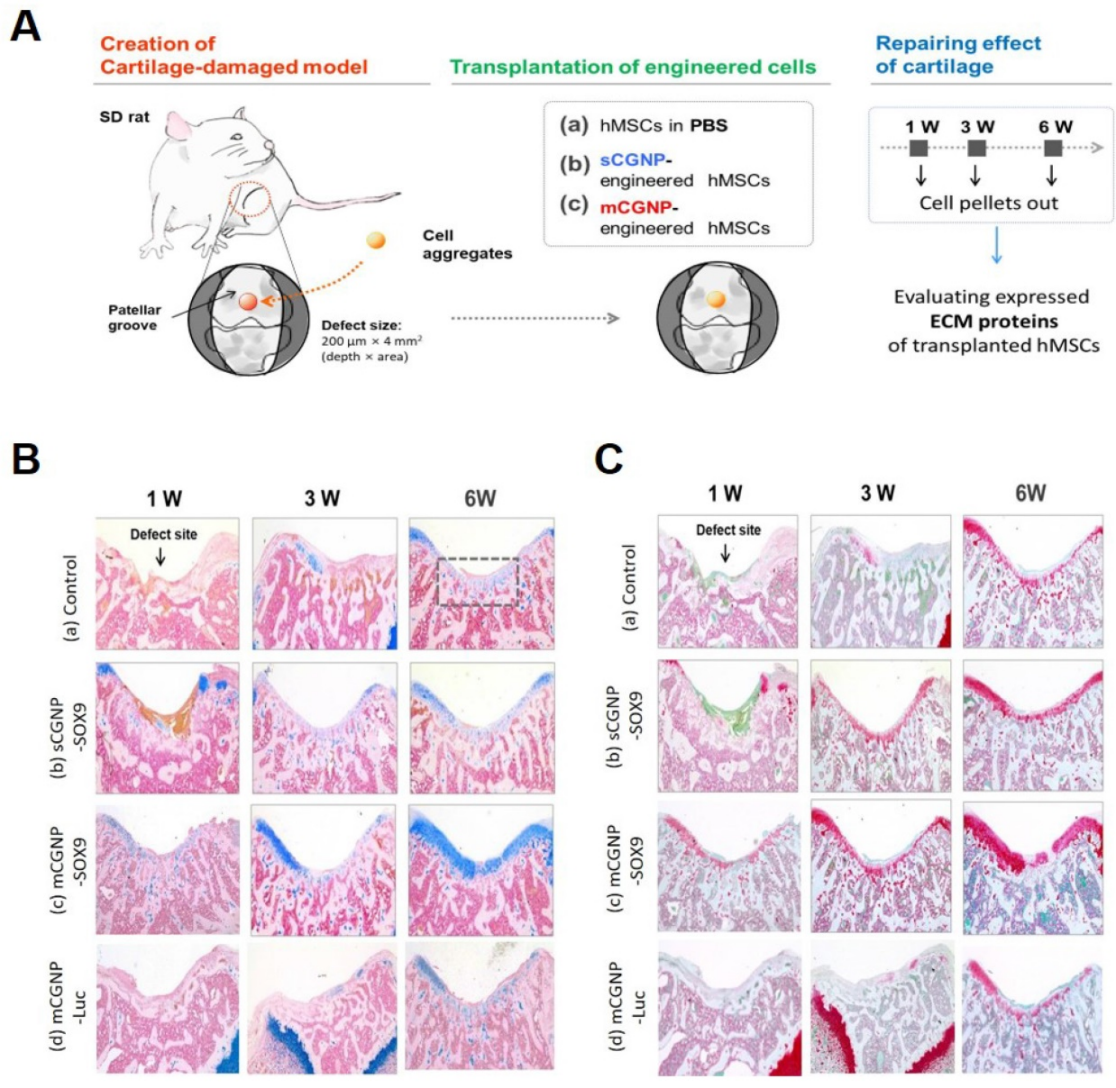
**Effect of mCGNP containing pDNA-SOX9 in the rat model of articular cartilage defect.** Schematic of experimental procedures, including the creation of the cartilage defect model, transplantation of the engineered cells, and repair of cartilage (A). Histological analysis (Alcian blue (B) and Safranin O (C), which label proteoglycans.) of chondrogenic differentiation of rat knee at 1w, 3w, and 6w after treatment.

Finally, to confirm the therapeutic effect, we applied the mCGNP system to an animal model of articular cartilage defected using Sprague Dawley (SD) rats. A schematic of the experiment is provided in Figure 5A. The animals were randomized into three groups: non-treated hMSCs, pSOX9 coated sCGNP- treated hMSCs, pSOX9 coated mCGNP-treated hMSCs, and pLuc coated mCGNP-treated hMSCs. Repair of defects in the patellar groove started 1 week after transplantation of mCGNP-treated hMSCs, earlier than in animals that received non-treated or sCGNP-treated hMSCs (Figure S12A). Expression of COLII and AGG began 1 week after transplantation of mCGNP-treated hMSCs, as determined at the RNA level by RT-PCR (Figure S12B) and at the protein level by western blot analysis (Figure S12C). In addition, the knee in each group were collected, sectioned and stained with Alcian blue (Figure 5B) and Safranin O (Figure 5C), which label proteoglycans expressed in cartilage. As in RT-PCR and western blot results, repair of defects in the patellar groove started to express proteoglycan 1 week after transplantation of mCGNP-treated hMSCs, earlier than non-treated or sCGNP-treated hMSCs.

Based on these results of *in vitro* and *in vivo* system, we conclude that induction of prolonged SOX9 expression caused stem cells to undergo chondrogenic differentiation more rapidly. mCGNPs shows the possibility as a tool for cell therapy by gene delivery for the reason. First, they were biocompatible as a gene delivery carrier. AuNPs (~5nm) and CPEI, composed of mCGNPs, are dissociated from mCGNP and then slowly released in the cell by exocytosis or degradation. Secondly, the pDNA was well-delivered by the mCGNP system during relatively prolonged time, resulting in the improvement of the therapeutic effect. The factor which needs to be expressed for as long as possible for the therapeutic effect, like SOX9 in the process of chondrogenesis, could be applied in the mCGNP system.

## Supplementary Material

Supplementary materials and methods, figures.
