# Supplementary material for: Multiply clustered gold-based nanoparticles complexed with exogenous pDNA achieve prolonged gene expression in stem cells
Source: Theranostics. 2019 Jul 9;9(17):5009–19. doi: 10.7150/thno.34487 (PMC6691390; doi:10.7150/thno.34487)
Supplement: Supplementary file 1 — Supplementary materials and methods, figures. [file thnov09p5009s1.pdf]

Submitted to Theranostics
The Forum for Diagnostics, Imaging, and Therapy Research

Supporting Information

Multiply clustered gold-based nanoparticles complexed with exogenous pDNA achieve prolonged gene expression in stem cells

Se Won Yi[1], Ji Sun Park[1], Hye Jin Kim[1], Jung Sun Lee[1], Dae Gyun Woo[2], and Keun-Hong Park[1,*]

[1] Department of Biomedical Science, College of Life Science, CHA University, 6F, CHA Biocomplex, Sampyeong-Dong, Bundang-gu, Seongnam-si, 13488, Republic of Korea.

[2] CHA Advanced Research Institute, 7F, CHA Biocomplex, Sampyeong-Dong, Bundang-gu, Seongnam-si, 13488, Republic of Korea.

***Corresponding Author:** Prof. Keun-Hong Park (pkh0410@cha.ac.kr)

Materials and Methods

1. Characterization of sCGNPs and mCGNPs

The morphologies and elemental distributions of sCGNPs and mCGNPs were studied using AFM. Samples were placed on a slide, dried at room temperature, and imaged at 20 kV in contact mode using a NanoWizard II atomic force microscope (JPK Instruments). The distribution of elements (Au of AuNP, N of CPEI, and S of HP) was visualized by EDS element mapping. For detection, a transmission electron microscope (ARM200F, JEOL) was equipped with an EDS detector, X-Max^{N} 80T (Oxford Instruments, Abingdon, Oxfordshire, UK). Au (gold), N (nitrogen), and S (sulfur) were detected in the indicated regions. The operating parameters were as follows: acceleration voltage, 200 kV; collection time, 10 min per sample. Elemental images of sCGNPs and mCGNPs were analyzed using the Aztec software (Laird, London, UK). And the mCGNPs were confirmed via fourier transform infrared (FT-IR) spectra obtained with a PerkinElmer Spectrum Two (L160000U, PerkinElmer, MA, USA).

2. Evaluation of cellular uptake of sCGNPs and mCGNPs by hMSCs

hMSCs were treated with sCGNPs or mCGNPs for 4 hours. For FACS analysis, treated cells were detached with Trypsin/EDTA (Invitrogen) and suspended in 0.1% BSA. The cell suspension was analyzed by FACS (Guava Technologies); fluorescence signals were detected for 10,000 cells per sample. For membrane staining, sCGNP- or mCGNP-treated hMSCs were stained with lipophilic tracer, 3,3'-Dioctadecyloxacarbocyanine perchlorate (DIO, Sigma-Aldrich) and visualized by fluorescence confocal microscopy (LSM 880 META, Zeiss, Oberkochen, Germany). Gold in sCGNPs and mCGNPs was detected by cellular TEM analysis. hMSCs were treated with sCGNPs and mCGNPs for 4 hours. For each group, 1×10^{6} hMSCs were cultured in a 3D environment for 24 hours. Pellets were pre-fixed with 2.5 %

glutaraldehyde for 4 hours. After washing with 0.1 M PBS, they were post-fixed with 1 % osmium tetroxide (OsO4) for 1 hour, and then dehydrated in an ethanol series (60 %, 70 %, 90 %, and 100 % in that order). Dehydrated pellets were embedded in resin overnight and sectioned with 10 μm thick. Sections were applied onto carbon-coated copper grids and stained with uranyl acetate and lead citrate. Grids were imaged by TEM (H-7600, Hitachi).

3. Histological analysis (Safranin O and Alcian blue staining) of pellets

hMSCs treated with nothing, sCGNPs, or mCGNPs were cultured in a 3D system for 21 days, and then fixed for 1 hour with 4 % paraformaldehyde. The pellets were embedded in optimal cutting temperature compound (TISSUE-TEK 4583; Sakura Finetek Inc.) and frozen at -80 °C, and the frozen samples were sliced into sections (10 μm thick) using a cryotome (HM 525; Thermo Fisher Scientific) at -25 °C. To evaluate chondrogenic differentiation, samples were stained with Alcian blue (A3157, Sigma-Aldrich) and Safranin O (HT90432, Sigma-Aldrich).

4. ***In Vivo* imaging on mCGNPs vs. sCGNPs**

Six-week old BALB/c female mice were purchased from Clea Japan. Animal experiments were approved by the Animal Care Committee of CHA University. BALB/c female mice were divided into three groups. For group I, control group (n=5), 100 μL of non-transfected hMSCs pellets ($5x10^6$ cells) was injected into the back subcutis of mice. In group II (n=5), 100 μL of GFP-transfected hMSCs pellets ($5x10^6$ cells, using sCGNPs) was injected into the back subcutis of mice. In group III (n=6), 100 μL of GFP-transfected hMSCs pellets ($5x10^6$ cells, using mCGNPs) was injected into the back subcutis of mice. Bioluminescent signals

(BLS) were monitored for 2 min by using the IVIS imaging system (Xenogen Corp., Alameda, Calif.).

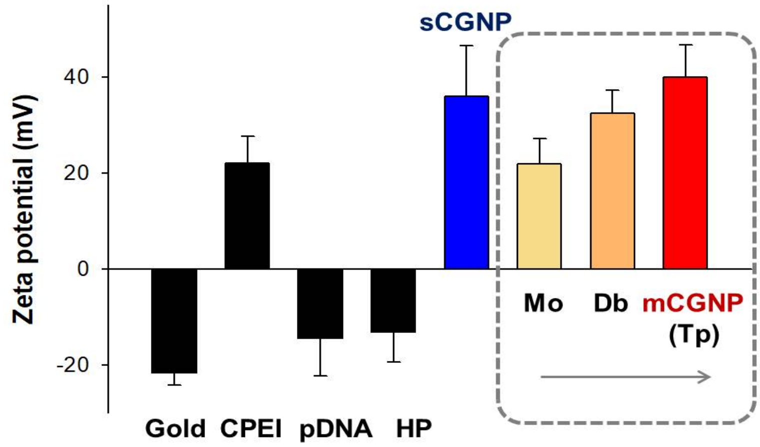

Figure S1. Surface charges of sCGNPs and mCGNPs, as determined by DLS.

Negatively charged AuNP, positively charged CPEI, negatively charged pDNA, and negatively charged HP yield sCGNPs and mCGNPs that are positively charged, and therefore suitable for endocytosis.

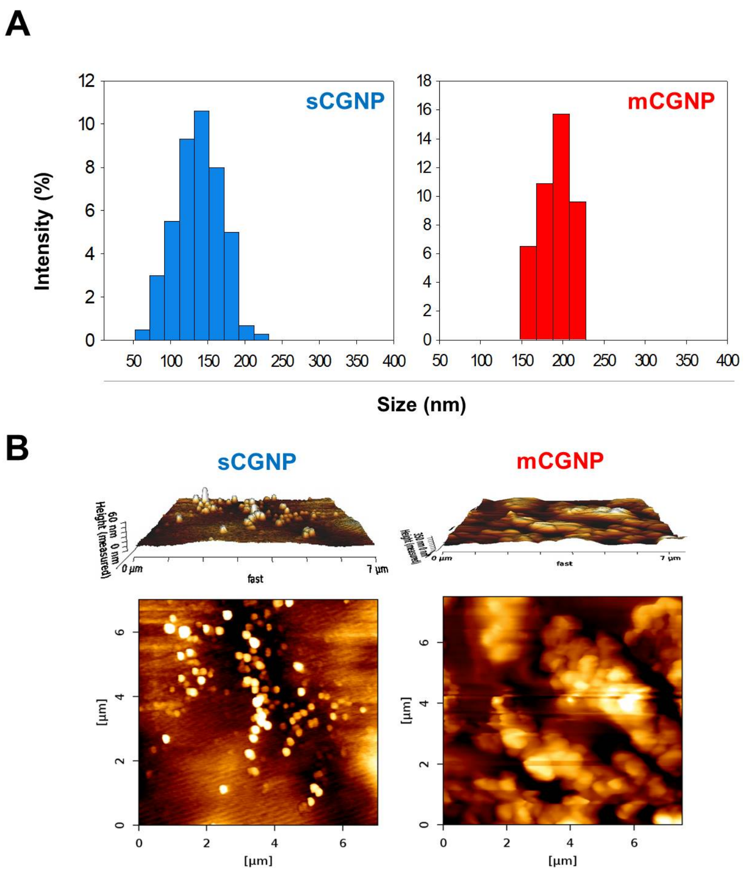

Figure S2. Morphologies and size distributions of sCGNPs and mCGNPs.

The size distribution of mCGNPs, as determined by dynamic laser scattering (DLS) **(A)** and atomic force microscopy (AFM) **(B)**, was more monodisperse than that of sCGNPs.

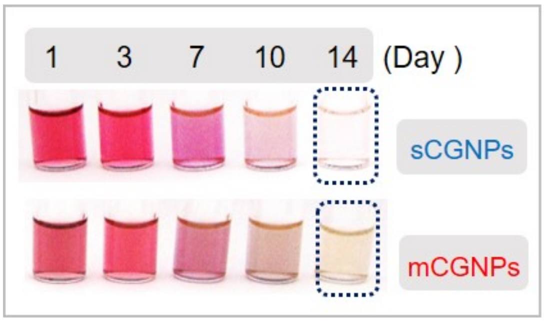

Figure S3. Photographs of sCGNPs and mCGNPs in aqueous solution as a function of time.

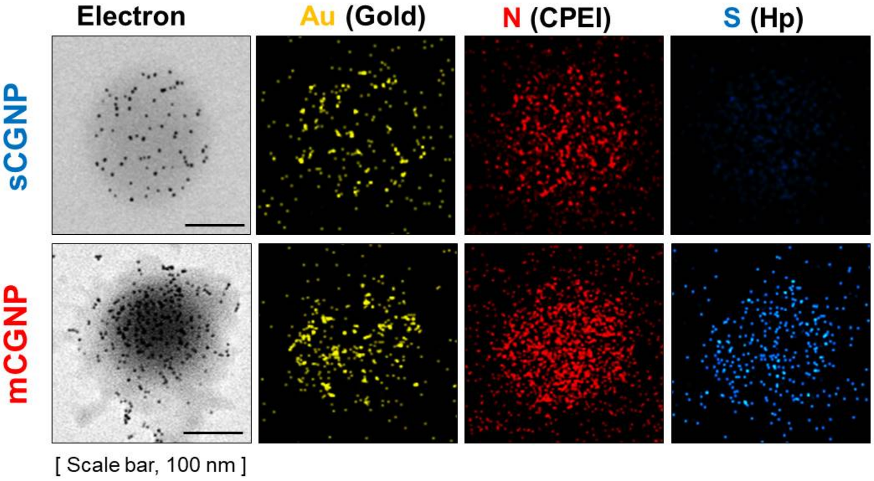

Figure S4. Elemental distribution (Au of AuNP, N of CPEI, and S of heparin) in sCGNPs and mCGNPs, as determined by energy-dispersive X-ray spectroscopy (EDS) element mapping.

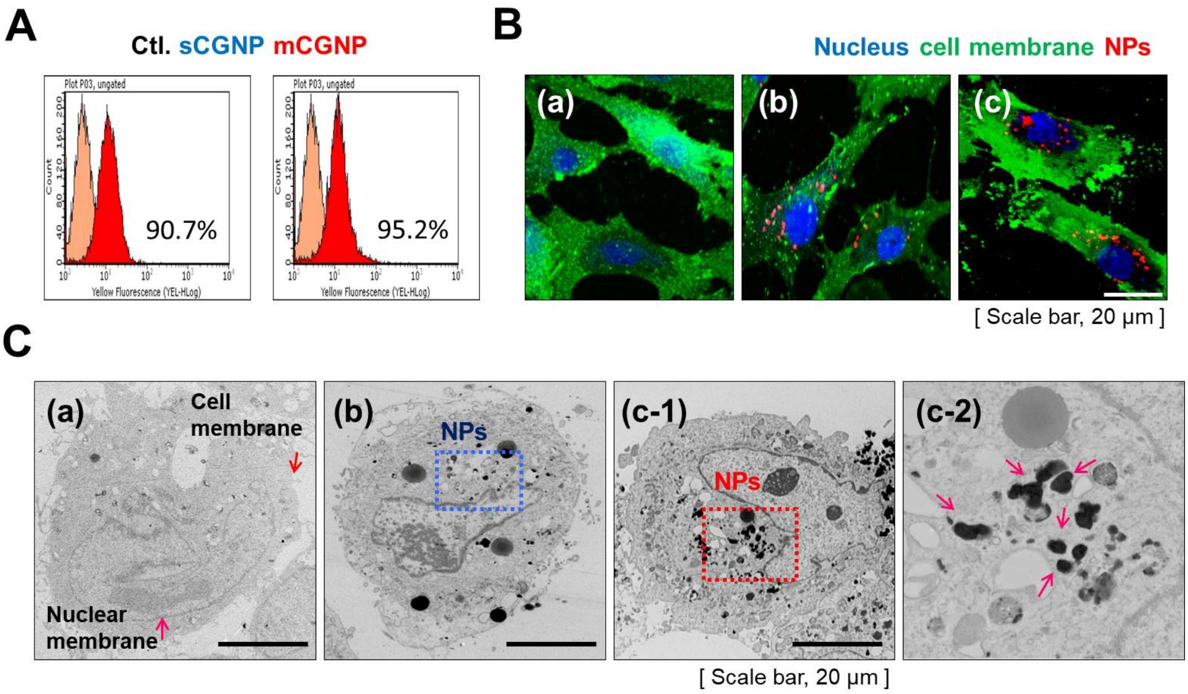

Figure S5. Cellular uptake of sCGNPs and mCGNPs to human mesenchymal stem cells (hMSCs). The cellular uptake efficiency of sCGNPs and mCGNPs was determined by FACS analysis **(A)**, membrane staining **(B)**, and cell TEM **(C)**.

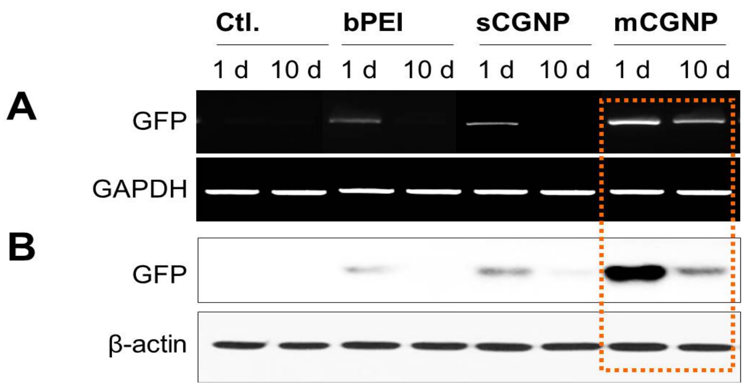

Figure S6. Verification of expression levels at 1 and 10 days after transfection of hMSCs using bPEI, sCGNPs, and mCGNPs

Proportion of hMSCs expressing GFP 1 and 10 days after transfection using bPEI, sCGNPs, or mCGNPs, as determined by RT-PCR (**A**) and western blotting (**B**).

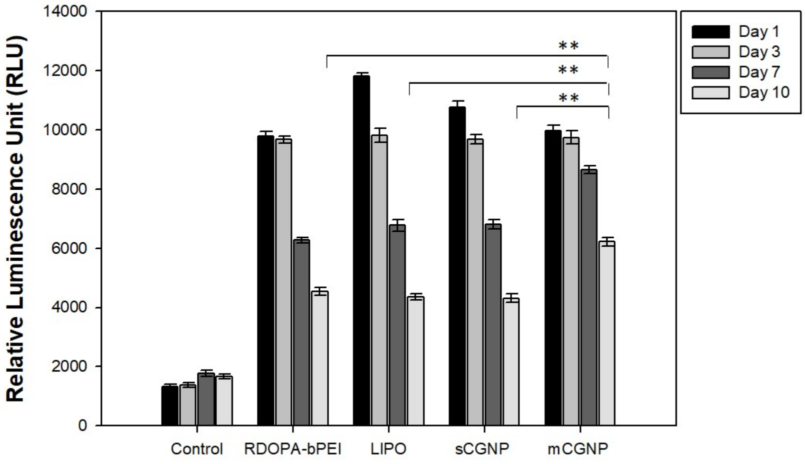

Figure S7. Verification of Luminoscence units (RLU) at 1, 3, 7 and 10 days after transfection of hMSCs using bPEI, Lipofectamine, sCGNPs, and mCGNPs. Each data represents the mean ±SD of relative luminescence unit (RLU) of three samples (n=3). (**: $p < 0.01$)

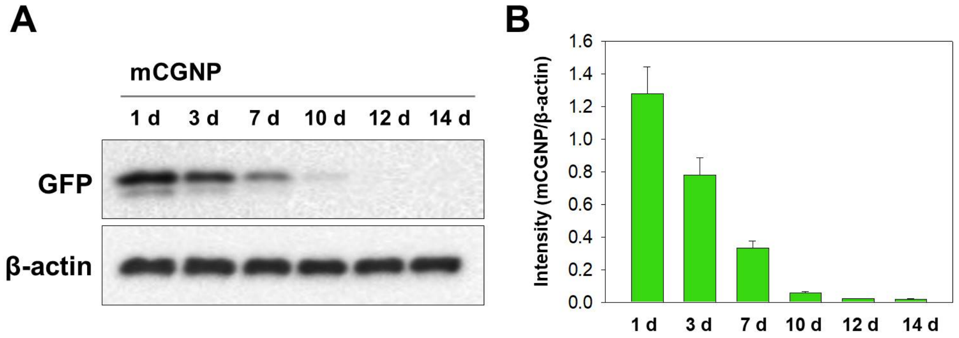

Figure S8. Determination of the duration of gene expression following transfection with mCGNPs.

Gene expression in hMSCs over 14 days following transfection of pDNA-EGFP using mCGNPs, evaluated by western blotting analysis **(A)** Band intensities are quantified in (**B**).

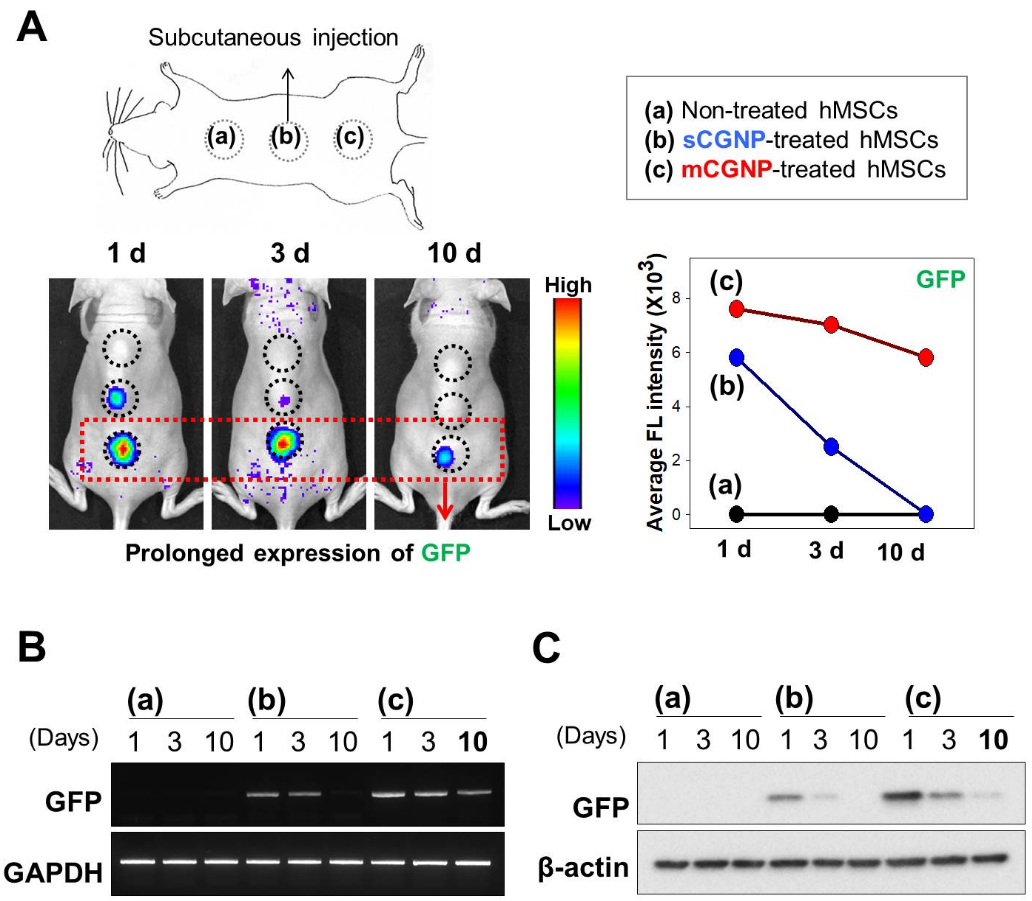

Figure S9. Expression of pDNA-EGFP on mCGNPs vs. sCGNPs *in vivo*

sCGNP- or mCGNP-treated hMSCs were cultured for 1, 3 or 10 days after transfection, and then GFP was detected using a Xenogen instrument **(A)**. The indicated groups of hMSCs were subcutaneously injected into BALB/c nude mice and detected using a Xenogen instrument. RNA levels of GFP in pelleted hMSCs from each mouse were determined by RT-PCR **(B)**. Protein levels of GFP from pellets were determined by western blot **(C)**.

(a) Non-treated hMSCs, (b) sCGNP-treated hMSCs, (c) mCGNP-treated hMSCs.

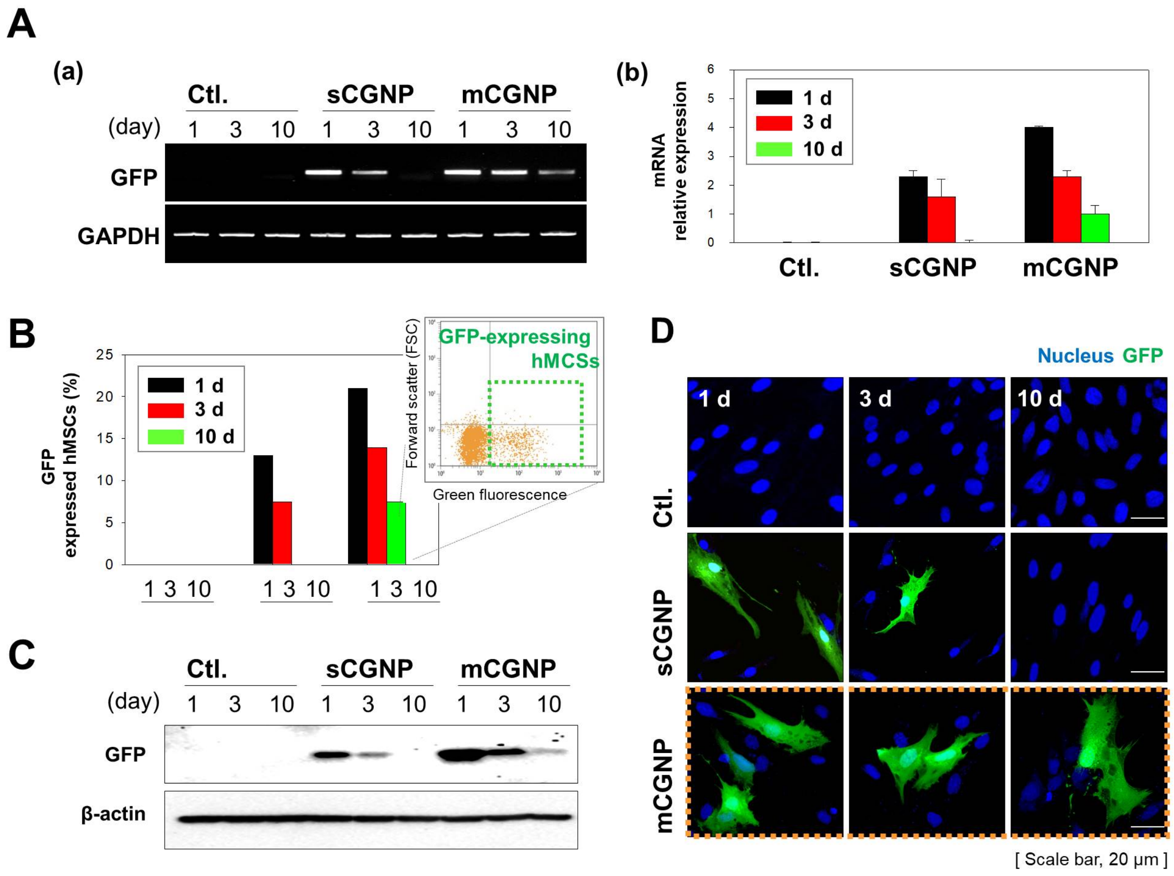

Figure S10. Expression of pDNA-EGFP on mCGNPs and sCGNPs for 10 days in hMSCs RNA level of GFP was determined by RT-PCR **(A)**. Protein level of GFP was determined by FACS **(B)**, western blot **(C)**, and confocal laser microscopy **(D)**.

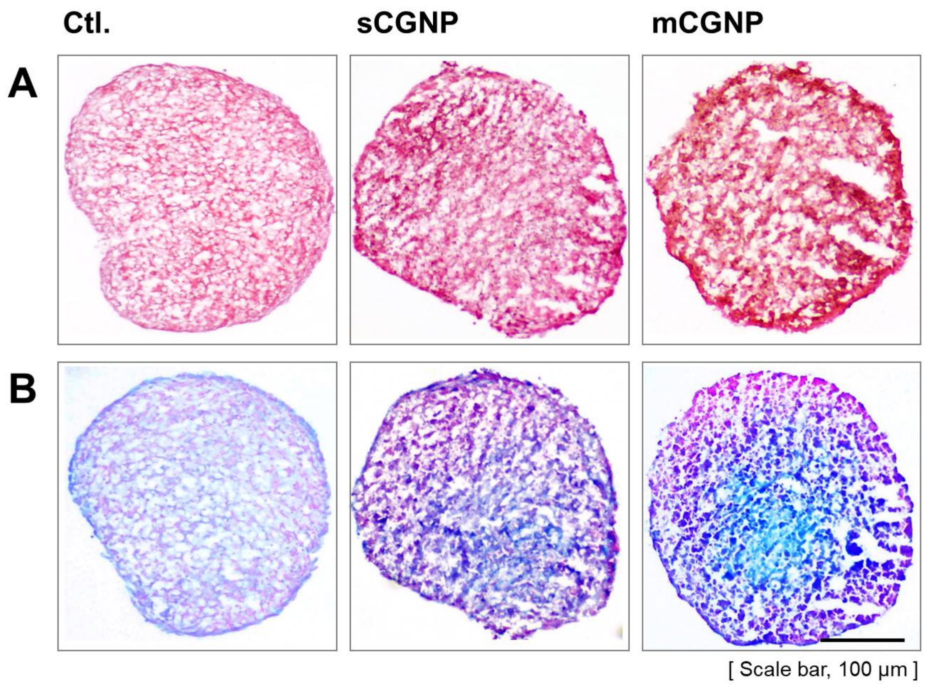

Figure S11. Histological analysis of chondrogenic differentiation of hMSCs transfected with nothing, sCGNPs, or mCGNPs complexed with pDNA-SOX9 over 21 days

hMSCs transfected with NPs were stained with Alcian blue (**A**) and Safranin O (**B**), which label proteoglycans.

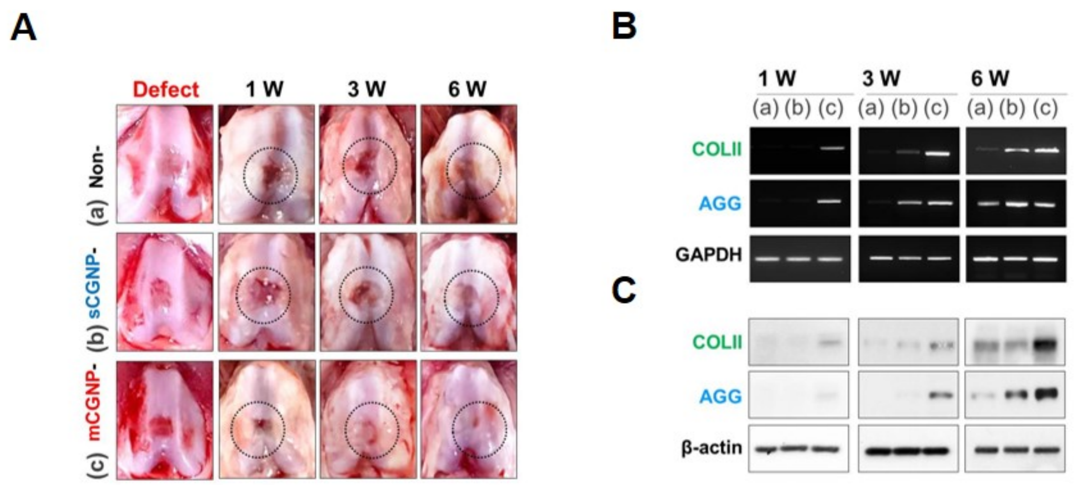

Figure S12. Effect of mCGNP containing pDNA-SOX9 in the rat model of articular cartilage defect.

Samples (transplanted pellets of engineered hMSCs and knee joints) were collected 1, 3, and 6 weeks after transplantation. Representative macroscopic observation images of rat knees are shown (A). Expression of ECM, including COLII and AGG, in pellets collected from defective cartilage was evaluated by RT-PCR (B) at the RNA level and western blot analysis (C).
